# Supplementary material for: Relationship between HLA genetic variations, COVID-19 vaccine antibody response, and risk of breakthrough outcomes
Source: Nat Commun. 2024 May 13;15:4031. doi: 10.1038/s41467-024-48339-5 (PMC11091043; doi:10.1038/s41467-024-48339-5)

Supplementary Materials

Supplementary table 1 Baseline characteristics of participants at the time of receiving the first dose of COVID-19 vaccine in the prospective cohort. 2
Supplementary table 2 Baseline characteristics of HLA DQB1*06 carriers and non-carriers in the cross-sectional cohort 3
Supplementary table 3 HLA alleles associated with COVID-19 vaccine antibody response in both discovery and validation datasets 4
Supplementary table 4 Association of the HLA genetic scores with COVID-19 vaccine antibody response 5
Supplementary table 5 The associations between individual HLA alleles, composite HLA genetic scores, and breakthrough COVID-19 outcomes 6
Supplementary table 6 Sensitivity analysis of Mendelian randomisation estimation 7

Supplementary figure 1 Manhattan plot of the genome-wide association study for COVID-19 vaccine antibody response 8
Supplementary figure 2 HLA associations with COVID-19 vaccine antibody response among White and other ethnic participants..9
Supplementary figure 3 Distribution of the HLA genetic scores for COVID-19 vaccine antibody response...10

Supplementary table 1 Baseline characteristics of participants at the time of receiving the first dose of COVID-19 vaccine in the prospective cohort.

	All participants
Number of recipients	357,806
Age, mean (SD)	69.28 (7.99)
Sex, n (%)	
Female	197824 (55.3)
Male	159982 (44.7)
Ethnicity	
White	328557 (91.8)
Other ethnic groups	29249 (8.2)
Vaccine types, n (%)[1]	
ChAdOx1	135237 (37.8)
BNT162b2	83202 (23.3)
Unknown	139367 (39.0)

Supplementary table 2 Baseline characteristics of HLA DQB1*06 carriers and non-carriers in the cross-sectional cohort

	1st-dose recipients			2nd-dose recipients		
	DQB1*06 carriers	DQB1*06 non-carriers	SMD	DQB1*06 carriers	DQB1*06 non-carriers	SMD
Number	61984	45191		39317	28508	
Age, mean (SD)	67.26 (7.59)	67.28 (7.59)	0.002	71.19 (6.56)	71.26 (6.56)	0.011
Age category, n (%)						
50-59 years	24865 (40.1)	18007 (39.8)	0.007	26592 (67.6)	19339 (67.8)	0.009
60-69 years	11567 (18.7)	8403 (18.6)		2985 (7.6)	2100 (7.4)	
>=70 years	25552 (41.2)	18781 (41.6)		9740 (24.8)	7069 (24.8)	
Sex, n (%)						
Female	34590 (55.8)	25171 (55.7)	0.002	22879 (58.2)	16538 (58.0)	0.004
Male	27394 (44.2)	20020 (44.3)		16438 (41.8)	11970 (42.0)	
Ethnicity						
White	52372 (84.5)	38139 (84.4)	0.003	33007 (84.0)	23807 (83.5)	0.012
Others	9612 (15.5)	7052 (15.6)		6310 (16.0)	4701 (16.5)	
Weeks since the latest vaccination, n (%)						
1 week	988 (1.6)	689 (1.5)	0.021	7609 (19.4)	5613 (19.7)	0.024
2 weeks	1709 (2.8)	1248 (2.8)		7448 (18.9)	5463 (19.2)	
3 weeks	2613 (4.2)	1996 (4.4)		6205 (15.8)	4510 (15.8)	
4 weeks	4051 (6.5)	3002 (6.6)		5198 (13.2)	3741 (13.1)	
5 weeks	5286 (8.5)	3902 (8.6)		4074 (10.4)	2944 (10.3)	
6 weeks	6040 (9.7)	4449 (9.8)		2899 (7.4)	2090 (7.3)	
7 weeks	7192 (11.6)	5158 (11.4)		2030 (5.2)	1396 (4.9)	
8 weeks	8611 (13.9)	6229 (13.8)		1447 (3.7)	991 (3.5)	
9 weeks	8984 (14.5)	6629 (14.7)		1006 (2.6)	712 (2.5)	
10 weeks	8552 (13.8)	6103 (13.5)		729 (1.9)	546 (1.9)	
11 weeks	6511 (10.5)	4808 (10.6)		433 (1.1)	300 (1.1)	
12 weeks	1447 (2.3)	978 (2.2)		239 (0.6)	202 (0.7)	
Vaccine types, n (%)[1]						
ChAdOx1	23039 (37.2)	16909 (37.4)	0.012	10824 (27.5)	7754 (27.2)	0.022
BNT162b2	8893 (14.3)	6295 (13.9)		10316 (26.2)	7271 (25.5)	
Unknown	30052 (48.5)	21987 (48.7)		18177 (46.2)	13483 (47.3)	

SMD, standardised mean difference

[1] Calculated based on a subgroup of participants who had complete linkage to primary care records for the information on COVID-19 vaccine types. The *P* value was calculated based on the Wald test.

Supplementary table 3 HLA alleles associated with COVID-19 vaccine antibody response in both discovery and validation datasets

Significant HLA alleles[1]	Discovery dataset			Validation dataset			Association direction
	Coefficient	Crude *P* value	FDR-corrected *P* value	Coefficient	Crude *P* value	FDR-corrected *P* value	
Antibody response to the 1st-dose vaccination							
DQB1*06:04	0.213	4.2×10^{-12}	1.1×10^{-10}	0.384	5.5×10^{-5}	2.6×10^{-3}	Positive
DRB1*13:02	0.203	1.9×10^{-14}	7.5×10^{-13}	0.305	2.5×10^{-4}	5.2×10^{-3}	Positive
DRB3*03:01	0.204	6.4×10^{-15}	4.3×10^{-13}	0.275	9.5×10^{-4}	7.4×10^{-3}	Positive
DRB3*01:01	0.137	2.8×10^{-22}	$5.6*\times10^{-20}$	0.109	$1.4*\times10^{-2}$	$4.7*\times10^{-2}$	Positive
DRB1*03:01	0.110	1.4×10^{-13}	4.7×10^{-12}	0.146	1.6×10^{-3}	9.4×10^{-3}	Positive
DQB1*02:01	0.108	3.2×10^{-13}	9.1×10^{-12}	0.144	1.9×10^{-3}	9.7×10^{-3}	Positive
DQA1*01:02	0.077	1.3×10^{-8}	2.1×10^{-7}	0.139	7.8×10^{-4}	7.3×10^{-3}	Positive
DRB1*15:01	0.058	1.6×10^{-4}	1.1×10^{-3}	0.119	1.1×10^{-2}	4.6×10^{-2}	Positive
DRB5*01:01	0.055	3.1×10^{-4}	1.9×10^{-3}	0.116	1.4×10^{-2}	4.7×10^{-2}	Positive
DQB1*05:01	-0.095	1.6×10^{-8}	2.3×10^{-7}	-0.170	1.4×10^{-3}	9.3×10^{-3}	Negative
DRB1*01:01	-0.078	3.8×10^{-5}	2.9×10^{-4}	-0.175	4.0×10^{-3}	1.9×10^{-2}	Negative
DQA1*01:01	-0.122	9.3×10^{-15}	4.7×10^{-13}	-0.180	3.3×10^{-4}	5.2×10^{-3}	Negative
C*16:01	-0.117	1.4×10^{-5}	1.1×10^{-4}	-0.216	1.2×10^{-2}	4.7×10^{-2}	Negative
DPB1*10:01	-0.157	3.1×10^{-4}	1.9×10^{-3}	-0.519	6.4×10^{-4}	7.3×10^{-3}	Negative
Antibody response to the 2nd-dose vaccination							
A*03:01	0.081	3.0×10^{-6}	7.7×10^{-5}	0.168	1.2×10^{-3}	1.5×10^{-2}	Positive
DQA1*01:01	-0.137	7.6×10^{-16}	1.5×10^{-13}	-0.165	1.2×10^{-3}	1.5×10^{-2}	Negative

[1]The alleles are ordered by the coefficient of the logistic regression model in the discovery dataset. FDR: false discovery rate. The *P* value was calculated based on the Wald test.

Supplementary table 4 Association of the HLA genetic scores with COVID-19 vaccine antibody response

Category	1st-dose antibody response			2nd-dose antibody response		
	Genetic score 1	Genetic score 2	Genetic score 3	Genetic score 1	Genetic score 2	Genetic score 3
Continuous*	1.14 (1.13 to 1.16)	1.16 (1.14 to 1.18)	1.17 (1.15 to 1.18)	1.10 (1.08 to 1.11)	1.12 (1.10 to 1.14)	1.14 (1.12 to 1.15)
Quintile 1	1 ref	1 ref	1 ref	1 ref	1 ref	1 ref
Quintile 2	1.19 (1.14 to 1.25)	1.17 (1.12 to 1.23)	1.16 (1.11 to 1.22)	1.11 (1.06 to 1.17)	1.13 (1.07 to 1.18)	1.09 (1.04 to 1.14)
Quintile 3	1.26 (1.21 to 1.32)	1.28 (1.22 to 1.34)	1.30 (1.24 to 1.36)	1.14 (1.09 to 1.20)	1.18 (1.12 to 1.24)	1.21 (1.15 to 1.27)
Quintile 4	1.37 (1.30 to 1.43)	1.37 (1.31 to 1.44)	1.38 (1.32 to 1.45)	1.29 (1.23 to 1.36)	1.26 (1.20 to 1.32)	1.26 (1.20 to 1.32)
Quintile 5	1.47 (1.41 to 1.54)	1.52 (1.45 to 1.59)	1.54 (1.47 to 1.61)	1.28 (1.22 to 1.34)	1.38 (1.32 to 1.46)	1.39 (1.32 to 1.46)

Supplementary table 5 The associations between individual HLA alleles, composite HLA genetic scores, and breakthrough COVID-19 outcomes

	Hazard ratio (95% CI)	
	**COVID-19 infection**	**Severe COVID-19**
Individual HLA alleles		
A*03:01	0.97 (0.94 to 1.00)	0.96 (0.86 to 1.07)
C*16:01	0.98 (0.93 to 1.03)	0.99 (0.82 to 1.20)
DPB1*10:01	1.07 (0.99 to 1.16)	1.00 (0.74 to 1.35)
DQA1*01:01	1.03 (1.00 to 1.06)	1.10 (0.99 to 1.22)
DQA1*01:02	0.98 (0.96 to 1.01)	0.95 (0.86 to 1.05)
DQB1*06:04	0.94 (0.88 to 1.01)	0.90 (0.70 to 1.15)
DRB3*01:01	0.98 (0.95 to 1.01)	0.91 (0.80 to 1.00)
Continuous genetic score for the 1st-dose antibody response	1.02 (0.99 to 1.05)	0.89 (0.82 to 0.98)
Continuous genetic score for the 2nd-dose antibody response	0.94 (0.90 to 0.98)	0.87 (0.76 to 0.99)
Categorical genetic score for the 1st-dose antibody response		
Reference: the highest 25%-100%	1	1
The lowest 25%	NC	1.06 (0.94 to 1.20)
The lowest 10%	NC	1.16 (0.97 to 1.37)
The lowest 5%	NC	1.26 (1.01 to 1.58)
Categorical genetic score for the 2nd-dose antibody response		
Reference: the highest 25%-100%	1	1
The lowest 25%	1.04 (1.01 to 1.08)	1.05 (0.93 to 1.19)
The lowest 10%	1.05 (1.00 to 1.11)	1.16 (0.98 to 1.38)
The lowest 5%	1.05 (0.98 to 1.12)	1.34 (1.08 to 1.67)

NC, Not calculated due to the lack of statistical significance for the continuous genetic score.

Supplementary table 6 Sensitivity analysis of Mendelian randomisation estimation

Exposure	Methods	Number of HLA alleles	COVID-19 infection	Severe COVID-19
1st-dose antibody response	MR Egger	24	11.19 (-5.39 to 25.16)	52.82 (14.14 to 74.08)
	Weighted median	24	14.69 (5.95 to 22.62)	46.90 (25.50 to 62.15)
	Inverse-variance weighted	24	14.51 (8.38 to 20.23)	41.13 (24.64 to 54.00)
	Simple mode	24	17.73 (4.69 to 28.98)	46.10 (11.97 to 67.00)
	Weighted mode	24	15.20 (3.64 to 25.38)	47.39 (20.86 to 65.03)
2nd-dose antibody response	MR Egger	4	-2.06 (-139.77 to 56.56)	64.88 (-622.50 to 98.29)
	Weighted median	4	20.08 (5.35 to 32.52)	49.51 (10.11 to 71.64)
	Inverse-variance weighted	4	21.34 (9.29 to 31.78)	46.56 (12.09 to 67.51)
	Simple mode	4	20.46 (0.81 to 36.22)	48.91 (-12.29 to 76.75)
	Weighted mode	4	19.58 (2.14 to 33.92)	50.07 (2.28 to 74.49)

Supplementary figure 1 Manhattan plot of the genome-wide association study for COVID-19 vaccine antibody response

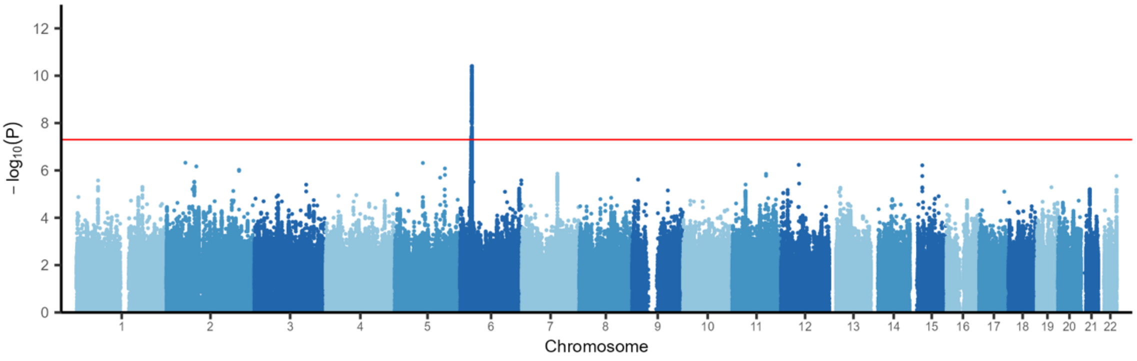

Antibody response to the 1st-dose

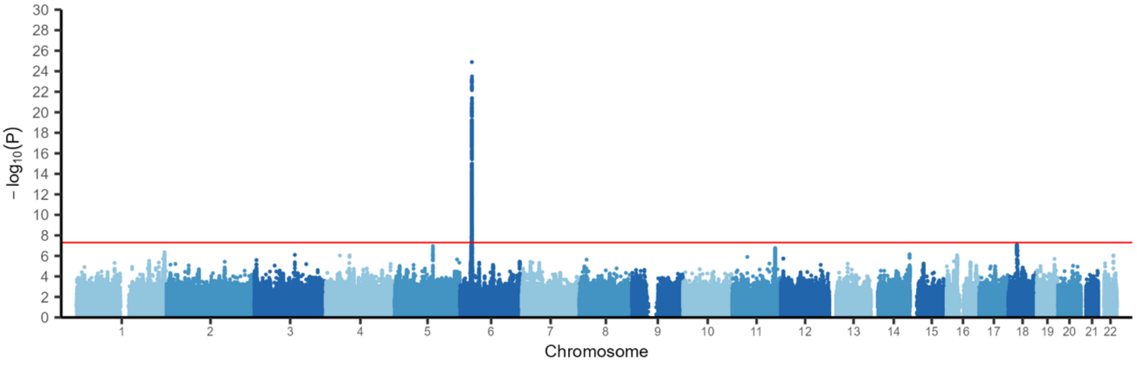

Antibody response to the 2nd-dose

Supplementary figure 2 HLA associations with COVID-19 vaccine antibody response among White and other ethnic participants

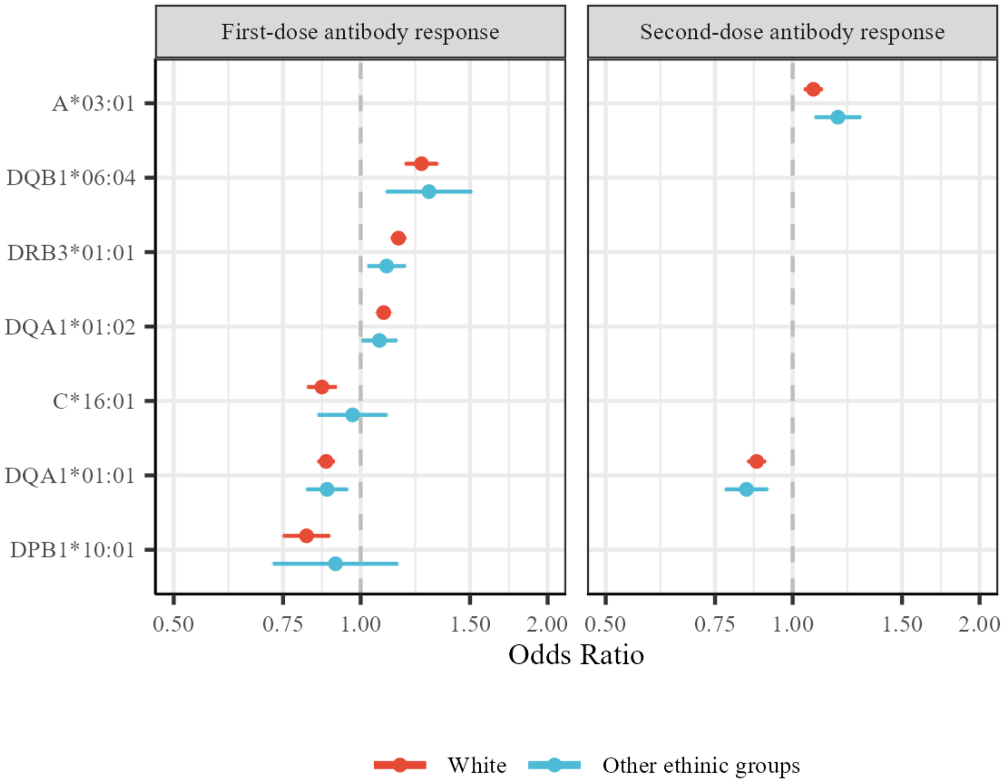

Supplementary figure 3 Distribution of the HLA genetic scores for COVID-19 vaccine antibody response

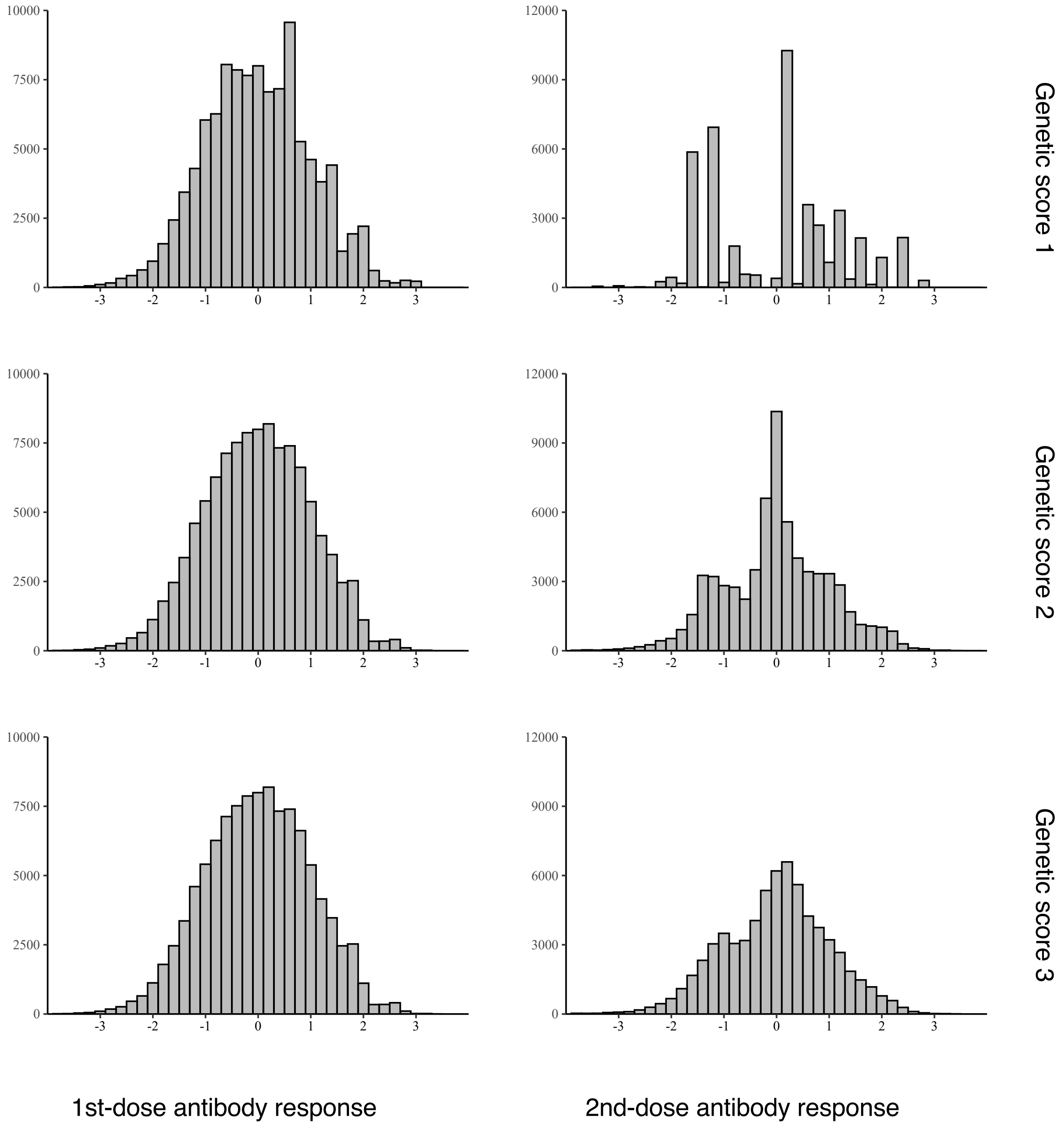

Supplement: Supplementary file 1 — Supplementary Information [file 41467_2024_48339_MOESM1_ESM.pdf]
