# Supplementary material for: Relationship between HLA genetic variations, COVID-19 vaccine antibody response, and risk of breakthrough outcomes
Source: Nat Commun. 2024 May 13;15:4031. doi: 10.1038/s41467-024-48339-5 (PMC11091043; doi:10.1038/s41467-024-48339-5)
Supplement: Supplementary file 3 — Reporting Summary [file 41467_2024_48339_MOESM3_ESM.pdf]

nature portfolio

Corresponding author(s): Daniel Prieto-Alhambra

Last updated by author(s): Apr 7, 2024

Reporting Summary

Nature Portfolio wishes to improve the reproducibility of the work that we publish. This form provides structure for consistency and transparency in reporting. For further information on Nature Portfolio policies, see our Editorial Policies and the Editorial Policy Checklist.

Statistics

For all statistical analyses, confirm that the following items are present in the figure legend, table legend, main text, or Methods section.

n/a	Confirmed	
☐	☒	The exact sample size (*n*) for each experimental group/condition, given as a discrete number and unit of measurement
☒	☐	A statement on whether measurements were taken from distinct samples or whether the same sample was measured repeatedly
☐	☒	The statistical test(s) used AND whether they are one- or two-sided<br>*Only common tests should be described solely by name; describe more complex techniques in the Methods section.*
☐	☒	A description of all covariates tested
☐	☒	A description of any assumptions or corrections, such as tests of normality and adjustment for multiple comparisons
☐	☒	A full description of the statistical parameters including central tendency (e.g. means) or other basic estimates (e.g. regression coefficient) AND variation (e.g. standard deviation) or associated estimates of uncertainty (e.g. confidence intervals)
☐	☒	For null hypothesis testing, the test statistic (e.g. F, t, r) with confidence intervals, effect sizes, degrees of freedom and P value noted<br>*Give P values as exact values whenever suitable.*
☒	☐	For Bayesian analysis, information on the choice of priors and Markov chain Monte Carlo settings
☒	☐	For hierarchical and complex designs, identification of the appropriate level for tests and full reporting of outcomes
☒	☐	Estimates of effect sizes (e.g. Cohen's *d*, Pearson's *r*), indicating how they were calculated

Our web collection on statistics for biologists contains articles on many of the points above.

Software and code

Policy information about availability of computer code

Data collection	Data can be download directly from UK Biobank website (https://www.ukbiobank.ac.uk) by the registered researcher after the approval of access application. No software was used for the data collection.
Data analysis	Data analysis was performed using UK Biobank Research Analysis Platform and R statistical software (version 4.1.2). The code used in this study can be accessed via https://github.com/xjq8065524/HLA_COVID_Vax_Antibody_Response.

For manuscripts utilizing custom algorithms or software that are central to the research but not yet described in published literature, software must be made available to editors and reviewers. We strongly encourage code deposition in a community repository (e.g. GitHub). See the Nature Portfolio guidelines for submitting code & software for further information.

Data

Policy information about availability of data

All manuscripts must include a data availability statement. This statement should provide the following information, where applicable:

- Accession codes, unique identifiers, or web links for publicly available datasets
- A description of any restrictions on data availability
- For clinical datasets or third party data, please ensure that the statement adheres to our policy

Bonafide researchers can apply for access to individual-level source data from the UK Biobank at http://ukbiobank.ac.uk/register-apply/. The aggregated data supporting the findings of this study are available within the paper, its supplementary information, and source data files.

Research involving human participants, their data, or biological material

Policy information about studies with human participants or human data. See also policy information about sex, gender (identity/presentation), and sexual orientation and race, ethnicity and racism.

Reporting on sex and gender	Among all participants included, 55.8% and 44.2% were female and male respectively. The gender information was collected at the baseline recruitment where all people self-reported their gender using questionnaire.
Reporting on race, ethnicity, or other socially relevant groupings	Among all participants included, 84.5% were White and the remaining were from other ethnic groups, including Asian or Asian British, Black or Black British, Chinese, and unspecific/unknown ethnicity.
Population characteristics	We included 380,677 UK Biobank participants during the study period from 01/01/2021 to 30/09/2021. Detailed baseline characteristics overall and in subgroups were described above and Table 1 in the manuscript.
Recruitment	The UK Biobank is an ongoing community-based prospective cohort study, which recruited more than 500,000 participants out of 9.2 million adults aged 40-69 years in the UK who were identified from National Health Service and invited to participants (5.5% response rate). The baseline survey took place from 2006 to 2010 in 22 assessment centers.
Ethics oversight	Ethics approval for the UK Biobank was granted by the North West Multi-Centre Research Ethics Committee in 2006 and was updated regularly after that (https://www.ukbiobank.ac.uk/learn-more-about-uk-biobank/about-us/ethics). All participants provided informed written consent to take part in the study and be followed-up through linkage to health-related records. This study received ethical approval from the UKBB Ethics Advisory Committee (EAC) under application 65397.

Note that full information on the approval of the study protocol must also be provided in the manuscript.

Field-specific reporting

Please select the one below that is the best fit for your research. If you are not sure, read the appropriate sections before making your selection.

☒ Life sciences ☐ Behavioural & social sciences ☐ Ecological, evolutionary & environmental sciences

For a reference copy of the document with all sections, see nature.com/documents/nr-reporting-summary-flat.pdf

Life sciences study design

All studies must disclose on these points even when the disclosure is negative.

Sample size	The sample size was determined by the maximum number of participants in the UK Biobank during the study period in order to maximize statistical power.
Data exclusions	Scotland and Wales participants were excluded due to lack of linkage to the primary care data source . A few England participants died before the COVID-19 outbreak (March 1, 2020) were also excluded.
Replication	The study utilised discovery and validation datasets to confirm the findings.
Randomization	No randomization procedure was involved in this observational study. Potential confounding was controlled by the regression modeling.
Blinding	Blinding was not applicable as no intervention were applied to participants.

Reporting for specific materials, systems and methods

We require information from authors about some types of materials, experimental systems and methods used in many studies. Here, indicate whether each material, system or method listed is relevant to your study. If you are not sure if a list item applies to your research, read the appropriate section before selecting a response.

Materials & experimental systems

n/a	Involved in the study	
☒	☐	Antibodies
☒	☐	Eukaryotic cell lines
☒	☐	Palaeontology and archaeology
☒	☐	Animals and other organisms
☒	☐	Clinical data
☒	☐	Dual use research of concern
☒	☐	Plants

Methods

n/a	Involved in the study	
☒	☐	ChIP-seq
☒	☐	Flow cytometry
☒	☐	MRI-based neuroimaging
